# Comparison of Human Eukaryotic Translation Initiation Factors 5A1 and 5AL1: Identification of Amino Acid Residues Important for EIF5A1 Lysine 50 Hypusination and Its Protein Stability

**DOI:** 10.3390/ijms24076067

**Published:** 2023-03-23

**Authors:** Yu-Yao Wu, Gao-Qi Wu, Na-Li Cai, Yan-Ming Xu, Andy T. Y. Lau

**Affiliations:** Laboratory of Cancer Biology and Epigenetics, Department of Cell Biology and Genetics, Shantou University Medical College, Shantou 515041, China

**Keywords:** hypusine, EIF5A1, EIF5AL1, protein stability, migration, proliferation

## Abstract

The human eukaryotic translation initiation factor 5A (EIF5A) family consists of three members, namely EIF5A1, EIF5A2, and EIF5AL1. Recent studies have shown that the expression of EIF5As is related to many human diseases, such as diabetes, viral infection, central nervous system injury, and cancer. Among them, EIF5A1 plays different functions in various cancers, possibly as a tumor-suppressor or oncogene, while EIF5A2 promotes the occurrence and development of cancer. Yet, the biological function of EIF5AL1 is not being studied so far. Interestingly, although there are only three amino acid (at residues 36, 45, and 109) differences between EIF5A1 and EIF5AL1, we demonstrate that only EIF5A1 can be hypusinated while EIF5AL1 cannot, and EIF5AL1 has a tumor-suppressor-like function by inhibiting cell proliferation and migration. We also show that EIF5AL1 protein turnover is mediated through the proteasomal pathway, and EIF5AL1 protein turnover is much faster than that of EIF5A1, which may explain their differential protein expression level in cells. By engineering single and double mutations on these three amino acids, we pinpoint which of these amino acids are critical for hypusination and protein stability. The data of this work should fill in the gaps in EIF5As research and pave the way for future studies on EIF5AL1.

## 1. Introduction

**Figure 1:**
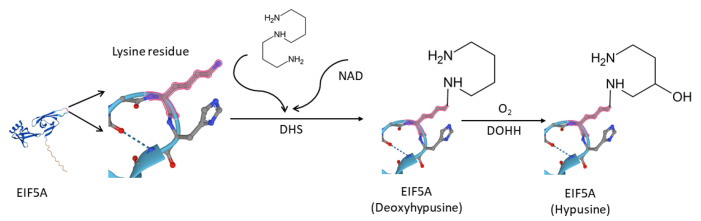
DHS catalyzes the transfer of the 4-aminobutyl moiety of spermidine to a specific lysine residue in the EIF5A precursor protein to form a deoxyhypusine-containing EIF5A intermediate, EIF5A (Deoxyhypusine) [11]. Subsequently, DOHH hydroxylates the aminobutyl portion of the deoxylated hypusine, resulting in the formation of EIF5A (Hypusine) [12].

In humans, the eukaryotic translation initiation factor 5A (EIF5A) family consists a total of three members, namely EIF5A1, EIF5A2, and EIF5AL1. EIF5A1 is the first protein demonstrated in eukaryotes that contains hypusine, i.e., N(epsilon)-(4-amino-2-hydroxybutyl)lysine, which is essential for the function of this family of proteins [1]. In 1971, scientists first discovered hypusine as a new amino acid occurring in bovine brain [2]. It was then later in 1983 that EIF5A1 being identified as the hypusine-containing protein [3]. As quite a rare type of post-translational modification, hypusination is catalyzed by deoxyhypusine synthase (DHS) and deoxyhypusine hydroxylase (DOHH) [4]. As Figure 1 shows, DHS catalyzes the transfer of the butyl portion of spermidine to the side chain of the lysine residue to form the deoxylated hypusine, which then DOHH hydroxylates the aminobutyl portion of the deoxylated hypusine, resulting in the formation of hypusine [4,5,6].

The EIF5A1 protein was initially identified as a translation initiation factor that promotes the first peptide bond formation during protein translation [7]. However, recent results suggest that the EIF5A1 protein also acts as a translation elongation factor [8]. Studies have shown that EIF5A1 is associated with a variety of human diseases, including diabetes, cancer, viral infection, and central nervous system injury. Meanwhile, research on EIF5A2 mainly focuses on the field of cancer, which shows that EIF5A2 can promote the development of a variety of cancers, such as bladder cancer, cervical cancer, and colorectal cancer [9]. The last member of the EIF5A family is a protein-coding gene named EIF5AL1, which is almost identical to the EIF5A1 protein, with differences in only three amino acids (AAs). Currently, the study of EIF5AL1 is next to nil, and only one study reported that EIF5AL1 was differentially expressed in patients with colorectal cancer metastasis [10].

Would the differences of these three AAs between EIF5A1 and EIF5AL1 lead to their differential biological functions? This study aims to investigate the importance of these three AAs on EIF5A1 and EIF5AL1 hypusine modification level and protein stability, as well as the associated phenotypic changes on cell growth and migration. Through the construction of EIF5A1 mutants, the impact of these three AAs is being studied for the first time, which might provide new insights into the differential roles of the EIF5A family in tumor suppression/promotion.

## 2. Results

### 2.1. EIF5AL1 Affects HeLa Cell Proliferation and Migration

**Figure 2:**
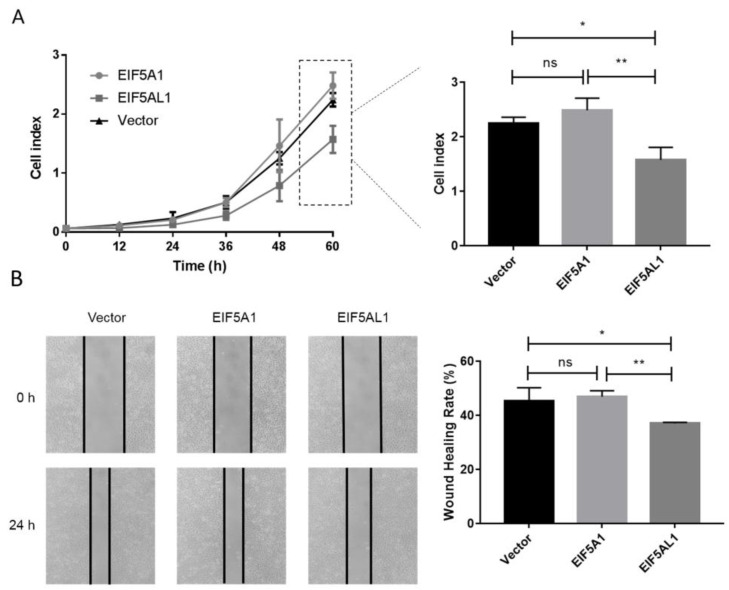
Overexpression of EIF5AL1 affects the proliferation and migration of HeLa cells. After transfecting and expressing EIF5A1, EIF5AL1, and vector plasmids in HeLa cells, respectively. (**A**) HeLa cells were seeded on 16-well E-plates with 3.0 × 10^3^ cells in each well and incubated in a cell incubator for 60 h and tested for cell index values over time. (**B**) HeLa cells were seeded into 12-well plates with 3.0 × 10^5^ cells per well for 24 h, and then, a wound healing assay was performed. ns, *p* > 0.05; *, *p* ≤ 0.05; **, *p* ≤ 0.01.

In order to investigate the function and molecular mechanisms of EIF5A1 and EIF5AL1 during cancer development and progression, we transfected the plasmids expressing EIF5A1 and EIF5AL1 in HeLa cells, respectively. Then, the proliferation capacity of the cells was determined by Real-Time Cell Analysis (RTCA) instrument, and the migratory ability of the cells was determined by the wound healing assay. The results showed that EIF5AL1 overexpression inhibited cell proliferation and the migration ability of HeLa cells, while EIF5A1 overexpression had no effect on the proliferation and migration ability of HeLa cells (Figure 2A,B).

### 2.2. Three Amino Acid Differences Exist between EIF5A1 and EIF5AL1

**Figure 3:**
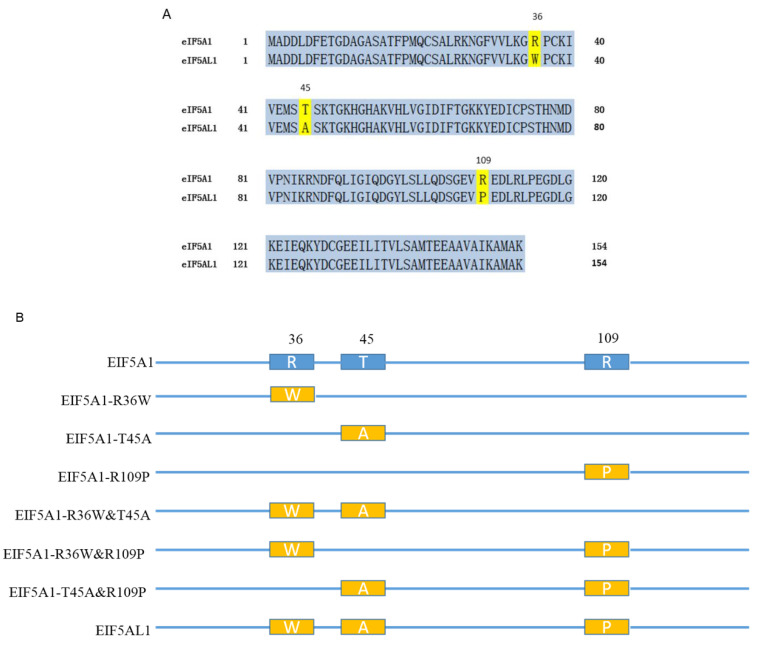
Comparison of the EIF5A1 and EIF5AL1 protein sequences. (**A**) Human EIF5A1 and EIF5AL1 protein sequences were downloaded from the UniProt database (https://www.uniprot.org/, accessed on 18 December 2019) with the ID numbers P63241 and Q6IS14, respectively. (**B**) Six EIF5A1 mutants were constructed based on the EIF5A1 and EIF5AL1 protein sequences.

Through the UniProt database, we found that both the EIF5A1 and EIF5AL1 proteins are composed of 154 AA residues, and their difference is only by three AAs, at positions 36, 45, and 109, respectively (Figure 3A). Thus, we further investigated why overexpression of EIF5A1 and EIF5AL1 proteins exhibited differential effects in HeLa cells.

Which of the three AAs in the EIF5A1 protein might lead to a different effect on the proliferation and migration of HeLa cells compared with the EIF5AL1 protein? We constructed six EIF5A1 mutant plasmids based on the EIF5A1 and EIF5AL1 protein sequences. As shown in Figure 3B, the EIF5A1-R36W, EIF5A1-T45A, and EIF5A1-R109P mutants only have one AA difference compared to the EIF5A1 protein sequence. In contrast, the EIF5A1-R36W&T45A, EIF5A1-R36W&R109P, and EIF5A1-T45A&R109P mutants have two AA differences from the EIF5A1 protein sequence, or just one AA difference from the EIF5AL1 protein.

### 2.3. Effects of the EIF5A1 Mutant Proteins on HeLa Cell Proliferation and Migration

**Figure 4:**
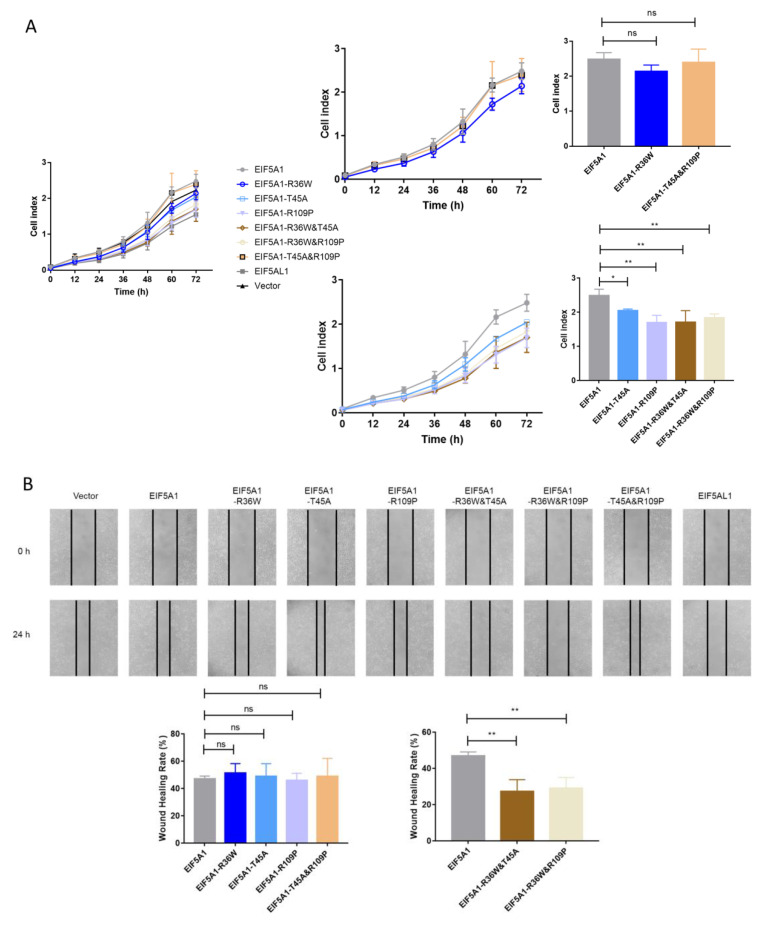
Overexpression of EIF5A1 mutants had different effects on HeLa cell proliferation and migration. After transfecting and expressing EIF5A1, EIF5AL1, EIF5A1 mutant, and vector plasmids in HeLa cells, respectively. (**A**) HeLa cells were seeded on 16-well E-plates with 3.0 × 10^3^ cells in each well and incubated in a cell incubator for 72 h, and tested for cell index values over time. (**B**) HeLa cells were seeded into 12-well plates with 3.0 × 10^5^ cells per well for 24 h, and then, a wound healing assay was performed. ns, *p* > 0.05; *, *p* ≤ 0.05; **, *p* ≤ 0.01.

Next, we studied the cell growth and migration by overexpressing EIF5A1 mutants in HeLa cells to find out which AA substitution in EIF5A1 and EIF5AL1 proteins caused their differential cellular effects. It was found that the transfection of the single-point mutants EIF5A1-T45A, EIF5A1-R109P and the double-point mutants EIF5A1-R36W&T45A, EIF5A1-R36W&R109P slowed down the cell growth rate (Figure 4A). However, overexpression of all single-point mutant proteins had no effect on HeLa cell migration ability compared with EIF5A1 overexpressing cells (Figure 4B). For the double-point mutants, the overexpression of EIF5A1-R36W&T45A and EIF5A1-R36W&R109P slowed down the rate of cell migration, with a statistically significant difference. Therefore, we believe that the effects of these three AAs on EIF5A1 protein function are not independent of each other but rather there are interactions and that various combinations have different effects. Notably, among all the EIF5A1 mutants, EIF5A1-R36W&T45A and EIF5A1-R36W&R109P reduced both the migration and proliferation of HeLa cells, which behave similarly to EIF5AL1.

### 2.4. The Hypusine Modification Levels of EIF5A1, EIF5AL1, and EIF5A1 Mutant Proteins

**Figure 5:**
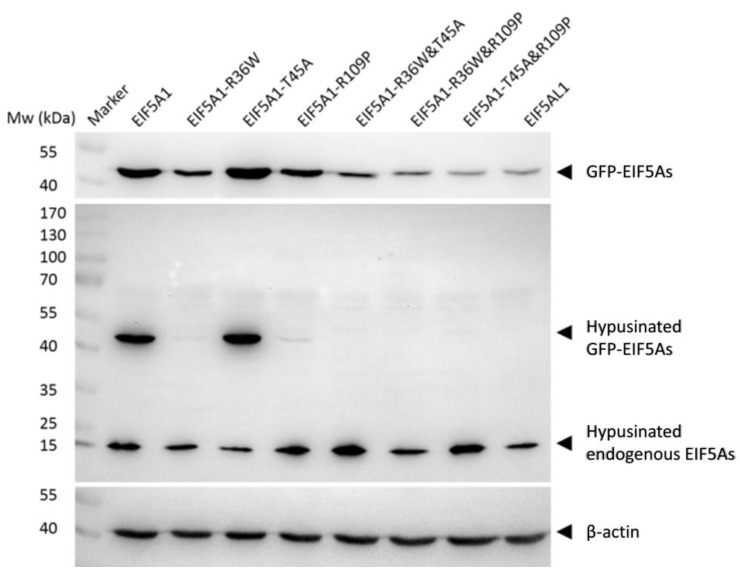
The hypusine modification levels of EIF5A1, EIF5AL1, and EIF5A1 mutant proteins. HeLa cells were transfected with EIF5A1, EIF5AL1 and EIF5A1 mutant plasmids, cells were harvested and lysed at 48 h after transfection. The proteins were extracted, and hypusine modification levels of EIF5A1, EIF5AL1, as well as EIF5A1 mutant proteins were determined by immunoblot analysis.

The hypusine modification of the EIF5A1 protein may directly affect the its function [13]. To further investigate why overexpression of EIF5A1 and EIF5AL1 in HeLa cells exhibited different phenotypes, we again transfected plasmids encoding EIF5A1, EIF5AL1, and EIF5A1 mutants into HeLa cells. Then, we examined the levels of hypusine modification of EIF5A1, EIF5AL1, as well as EIF5A1 mutant proteins. The results showed that the endogenous hypusine modification was present in the cells of all groups (which are populations of wildtype EIF5As expressed by the cells). For the ectopically-expressed EIF5As, except for the EIF5A1 protein and the EIF5A1-T45A mutant protein, the hypusination level of other proteins were barely detectable (Figure 5). This suggests that the AAs at positions 36 and 109 of EIF5A1 protein are important for the formation of its hypusine modification but not for the AA at position 45. Notably, EIF5A1 and EIF5A1-T45A with similar and robust levels of hypusine modification appeared to be more stable, as evidenced by their higher protein abundance as compared to EIF5AL1 and all the other EIF5A1 mutants.

### 2.5. The Protein Stability of EIF5A1, EIF5AL1, and EIF5A1 Mutants

**Figure 6:**
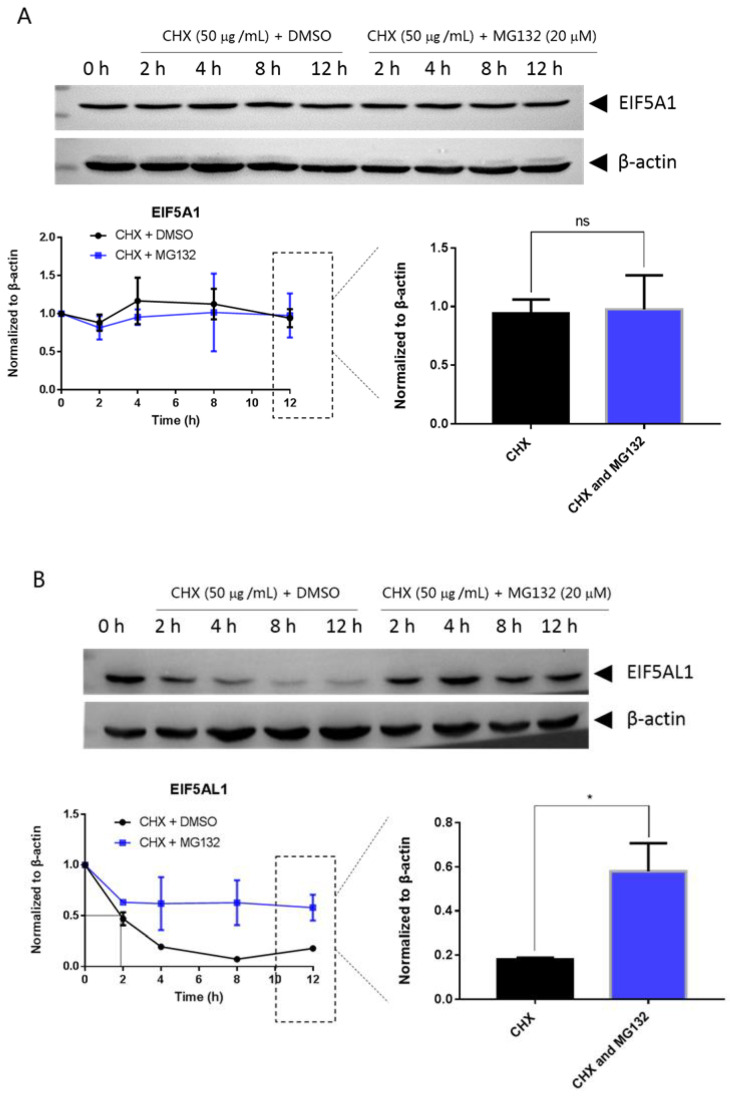

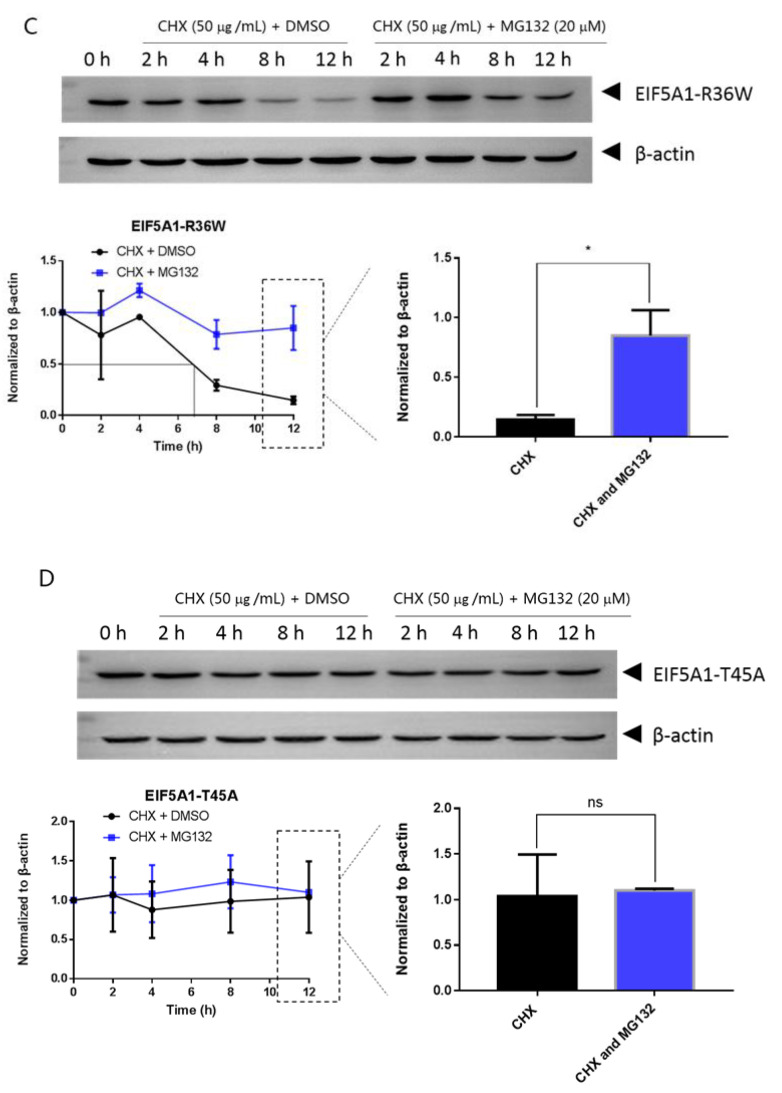

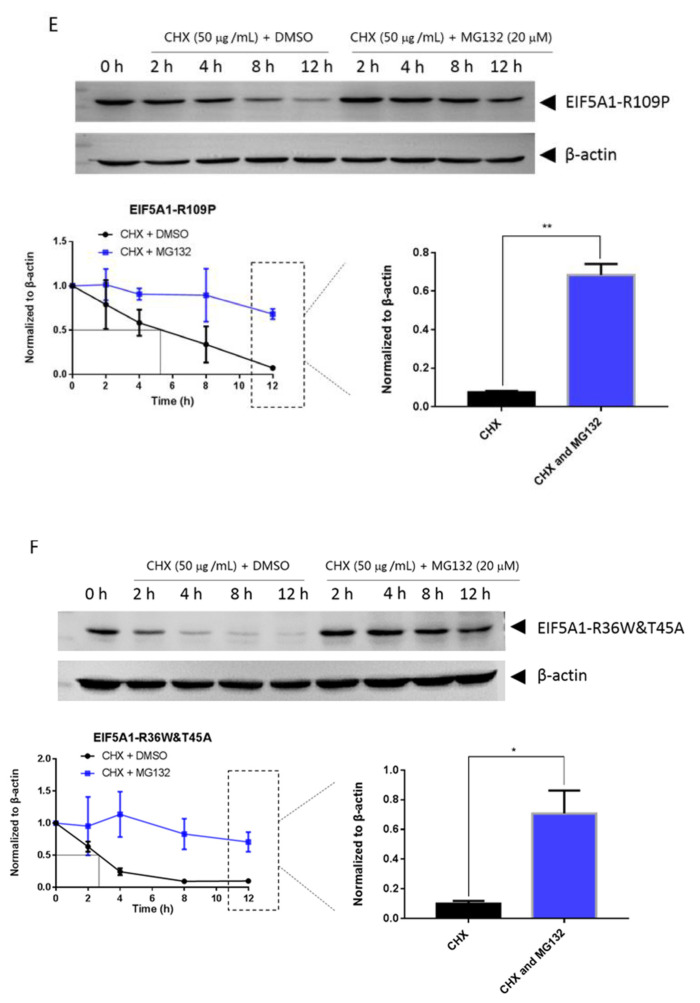

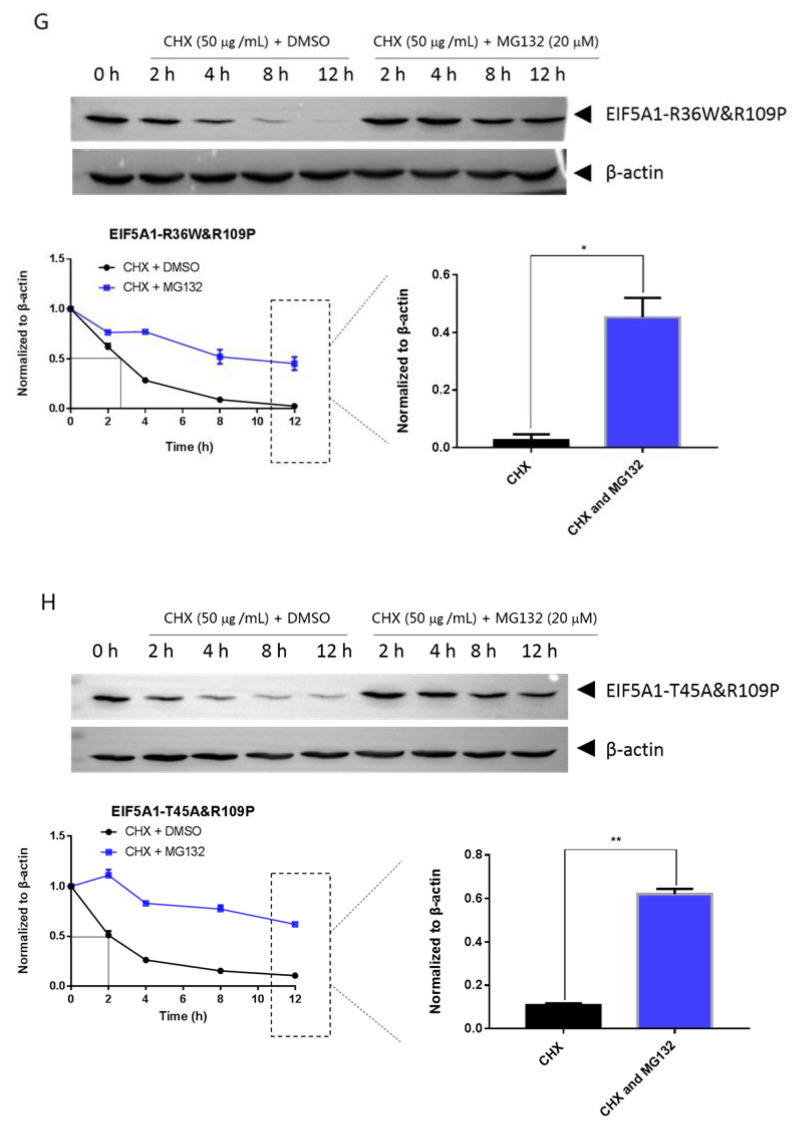
HeLa cells were seeded into 10 cm cell culture dishes for 24 h, transfected with EIF5A1, EIF5AL1, and EIF5A1 mutant plasmids, and the cells were averaged to nine 6 cm cell culture dishes at 24 h post-transfection. After 24 h, four of these cells were treated individually with CHX and four others with CHX and MG132. Cells were harvested after the indicated time points, and the abundance of EIF5A1, EIF5AL1, as well as EIF5A1 mutant proteins were determined by immunoblot analysis using GFP antibody. β-actin was used as the loading control. (**A**) EIF5A1 protein abundance. (**B**) EIF5AL1 protein abundance. (**C**) EIF5A1-R36W protein abundance. (**D**) EIF5A1-T45A protein abundance. (**E**) EIF5A1-R109P protein abundance. (**F**) EIF5A1-R36W&T45A protein abundance. (**G**) EIF5A1-R36W&R109P protein abundance. (**H**) EIF5A1-T45A&R109P protein abundance. ns, *p* > 0.05; *, *p* ≤ 0.05; **, *p* ≤ 0.01.

Mutations can affect not only protein structure but also protein stability and hence protein function [14]. We investigated the stability of EIF5A1, EIF5AL1, and EIF5A1 mutant proteins. We treated the cells with protein synthesis inhibitor cycloheximide (CHX) and found that the expression level of the EIF5A1 protein (Figure 6A) and the single-point mutant EIF5A1-T45A protein (Figure 6D) did not change significantly over time, while the EIF5AL1 (Figure 6B), EIF5A1-R36W (Figure 6C), EIF5A1-R109P (Figure 6E), EIF5A1-R36W&T45A (Figure 6F), EIF5A1-R36W&R109P (Figure 6G), and EIF5A1-T45A&R109P (Figure 6H) protein levels were rapidly decreased. Quantification of the signals revealed that the half-life of EIF5AL1 protein (Figure 6B) and EIF5A1-T45A&R109P protein (Figure 6H) were only 2 h, EIF5A1-R36W&T45A protein (Figure 6F) and EIF5A1-R36W&R109P protein (Figure 6G) were 3 h, EIF5A1-R109P protein (Figure 6E) was 5 h, EIF5A1-R36W protein (Figure 6C) was 7 h, whereas EIF5A1 protein (Figure 6A) and EIF5A1-T45A protein (Figure 6D) had half-life of more than 12 h. The above results indicate that the AAs at positions 36 and 109 of EIF5A1 protein are important for its stability. To further investigate the degradation pathway of these proteins, we inhibited the cellular proteasomal degradation pathway with proteasome inhibitor MG132 and found that cells cotreated with CHX and MG132 sustained their protein levels compared with CHX alone (Figure 6B,C,E,F,G,H), indicating that EIF5AL1, EIF5A1-R36W, EIF5A1-R109P, EIF5A1-R36W&T45A, EIF5A1-R36W&R109P and EIF5A1-T45A&R109P proteins may be degraded through the ubiquitin-proteasome pathway.

### 2.6. Three-Dimensional Modeling and Bioinformatic Analysis

Except for EIF5A1, currently, the crystal structures of other EIF5A1 mutants or EIF5AL1 are unavailable. Through the AlphaFold Protein Structure Database [15,16], we obtained the protein structures of EIF5A1 and EIF5AL1 (Figure S1). The results show that the mutation of the AA at position 109 resulted in an altered local folding of the protein, while the AA mutations at positions 36 and 45 do not appear to cause structural differences between EIF5A1 and EIF5AL1. Since the protein structure of the other mutants cannot be obtained in this database, therefore, the protein sequences of EIF5A1, EIF5AL1, and EIF5A1 mutants (EIF5A1-R36W, EIF5A1-T45A, EIF5A1-R109P, EIF5A1-R36W&T45A, EIF5A1-R36W&R109P, and EIF5A1-T45A&R109P) were sent to the D-I-TASSER server for three-dimensional protein modeling analysis [17]. In regard to protein structure modeling, the top five models with the highest estimated TM-score (eTM-score) were generated for each of the AA sequence submitted to D-I-TASSER (Figure S2). The results showed that the predicted structures of EIF5A1 were not significantly different from the mutants, as well as EIF5AL1.

## 3. Discussion

EIF5A1 can play different roles in cancer occurrence and development [18]. Researchers found that loss of EIF5A1 and AMD1 cooperate lymphoma progression in mice [19]. It has been shown that EIF5A1 overexpression promoted human lung cancer A549 cell apoptosis, with the upregulation of Bax and Bid, which may be the molecular mechanism of EIF5A1-mediated apoptosis [20]. On the other hand, in pancreatic ductal adenocarcinoma (PDAC), EIF5A1 mediates PDAC cell migration and invasion by modulating RhoA/ROCK protein expression levels while pharmacological inhibition or genetic knockdown of EIF5A1 reduces PDAC cell migration, invasion, and metastasis in vitro and in vivo [21]. However, to the best of our knowledge, little is known about the function of EIF5AL1. Our study showed that EIF5A1 protein overexpression had no effect on the cell proliferation or migration of HeLa cells, but EIF5AL1 overexpression inhibited the cell proliferation and migration of HeLa cells, indicating that EIF5AL1 may function as a tumor suppressor gene. There were only three AA differences between EIF5A1 protein and EIF5AL1 protein, and to investigate the effect of these three AAs on EIF5A1 protein function, six mutant plasmids were constructed, namely EIF5A1-R36W, EIF5A1-T45A, EIF5A1-R109P, EIF5A1-R36W&T45A, EIF5A1-R36W&R109P, and EIF5A1-T45A&R109P. Examination of these mutants showed that EIF5A1-R36W&T45A and EIF5A1-R36W&R109P proteins have similar functions to EIF5AL1 and significantly inhibited proliferation and migration of HeLa cells as compared to EIF5A1 protein.

As a unique post-translational modification of the EIF5A family proteins, hypusine plays a decisive role in the function of EIF5A proteins. The EIF5A precursor protein and its two modifying enzymes are highly conserved, suggesting that EIF5A and its hypusination has important cellular functions [22]. For example, the knockdown of DOHH catalyzing hypusine formation significantly inhibited cervical cancer cell proliferation [23]. Similarly, treatment of glioblastoma cell lines with GC7, a specific DHS-inhibitor, showed a strong antiproliferative effect in these cells, while normal human astrocytes were not affected [24]. Hypusination also affected the localization of EIF5A1 in cells, and EIF5A1 protein without hypusine modification was found to have no preference for localization in the cytoplasm and nucleus, while EIF5A1 protein with hypusine modification was found to be localized mainly within the cytoplasm [25,26,27,28]. The EIF5A1 protein is involved in the apoptotic pathway, and its overexpression is able to promote apoptosis. Interestingly, hypusine seems to have no effect on this function of EIF5A1 because either the EIF5A1 protein that can be modified to hypusine or a K50A mutant of EIF5A1 that cannot be modified to hypusine can promote apoptosis [20]. Our study shows that EIF5A1 protein has high level of hypusination but virtually undetectable level of hypusination on EIF5AL1 protein, which may be one reason for the differential effects of EIF5A1 and EIF5AL1 proteins on the proliferation and migration of HeLa cells. Meanwhile, examination of the hypusination level of the EIF5A1 mutants demonstrated that, except for EIF5A1-T45A, the hypusination of all other mutants were abolished, indicating that the AAs at positions 36 and 109 of the EIF5A1 protein are important for hypusine formation.

Since the EIF5A family proteins are the only proteins in cells with hypusine modification, the addition of radioactive spermidine followed by unlabelled spermidine can determine the protein turnover time in cell culture. Using this method, Gerner et al. measured the half-life of EIF5A1 protein. The experimental results showed that EIF5A1 protein in rat liver cancer cells was stable, with half-life of longer than 24 h [29]. In human HEK293T cells, the measured EIF5A1 protein half-life was 29.1 h [30]. Shang et al. showed that the EIF5A1 protein is degraded by the ubiquitin-proteasome pathway and that the E3 ubiquitin ligase CHIP is responsible for the polyubiquitination of the EIF5A1 protein [30]. The stability of intracellular EIF5A1 protein is also affected by extracellular stimulation. In human colon cancer cells, acute heat stress caused a striking loss of EIF5A1, as its half-life was changed from >20 h to less than 30 min [31]. The ubiquitin-proteasome pathway is the main pathway of intracellular protein degradation in cells, and studies show that the proteasome function involves basic cell biological functions, such as apoptosis, cell cycle control, DNA repair, stress response, gene transcription, and signal transduction [32]. Abnormal proteasome function is related to the occurrence of cancer, inflammation, and neurodegenerative diseases [33,34,35]. MG132 is the most commonly used inhibitor of the proteasome, and MG132 can permeabilize the cells and selectively inhibit proteasome, thereby inhibiting the protein degradation [36]. Our study shows that EIF5AL1 protein is extremely unstable compared to the long half-life of EIF5A1, being degraded by half in 2 h after CHX inhibited new protein synthesis, but cells treated together with CHX and MG132 had a significantly longer EIF5AL1 half-life, indicating that EIF5AL1 degradation occurs through the ubiquitin-proteasome pathway. Among the six EIF5A1 mutant proteins, the stability of EIF5A1-T45A was similar to that of EIF5A1, while EIF5A1-R36W, EIF5A1-R109P, EIF5A1-R36W&T45A, EIF5A1-R36W&R109P, and EIF5A1-T45A&R109P showed significantly lower stability than the EIF5A1 protein, indicating that the AAs at positions 36 and 109 of EIF5A1 protein are important for its stability.

In 2003, Thompson et al. found that the deletion of the C-terminus of EIF5A (∆90–152) completely eliminated the interaction between EIF5A and yeast DOHH [37]. By protein structure prediction analysis, we found that the mutation of AA at position 109 caused a significant change in the protein structure. Therefore, as long as EIF5A1 has a mutation of AA at position 109, it might not be hypusinated even with the presence of lysine 50. The mutation of the AA at position 36, although it does not seem to cause an obvious change in the protein structure, has affected the stability of the protein, which may explain the failure of EIF5A1-R36W to be hypusinated. For the fact that except for EIF5A1, the specific crystal structures of EIF5AL1 and EIF5A1 mutants were unstudied, therefore, here we can only predict their structures through the available online prediction software. However, there could be a divergence between the actual structure and the predicted structure, which might limit the results of the structure prediction of these mutant proteins through the database. Therefore, the structural changes caused by these AA mutations should be further explored in the future. Nevertheless, from the results we present here, we can conclude that mutation of the AAs at positions 36 and 109 in the EIF5A1 protein sequence will seriously affect the hypusine modification of the protein, but the specific mechanism still needs to be demonstrated by further experiments.

## 4. Materials and Methods

### 4.1. Cell Culture and Reagents

The human cervical cancer (HeLa) cell line was purchased from the American Type Culture Collection (ATCC) (Rockville, MD, USA). HeLa cells were routinely cultured in MEM medium containing 10% fetal bovine serum (FBS) and 1% penicillin/streptomycin at 37 °C in a 5% CO_2_ incubator as recommended by ATCC. All general reagents used in this study were obtained from GE Healthcare (Uppsala, Sweden) and Sigma-Aldrich (St. Louis, MO, USA).

### 4.2. Plasmids and Transfection

The pEGFP-N1 was purchased from CLONTECH Laboratories, Inc. (Palo Alto, CA, USA). The pEGFP-N1-EIF5A1 and pEGFP-N1-EIF5AL1 expression vectors were constructed by our laboratory members and stored at −20 °C. Site-directed mutagenesis of the plasmid was carried out using the Agilent QuikChange Lightning Site-Directed Mutagenesis Kit (Santa Clara, CA, USA). Mutagenesis primers were designed by using the online tool (www.agilent.com/genomics/qcpd, accessed on 18 December 2019) (Primers are shown in Table S1). We constructed six EIF5A1 mutant plasmids based on the EIF5A1 and the EIF5AL1 protein sequences, namely EIF5A1-R36W, EIF5A1-T45A, EIF5A1-R109P, EIF5A1-R36W&T45A, EIF5A1-R36W&R109P, and EIF5A1-T45A&R109P (Figure 3B). The authenticity of all the mutant plasmids was confirmed by DNA sequencing, and the sequencing results were shown in Table S2. Transfections were performed with PEI reagent, according to the manufacturer’s instructions.

### 4.3. Cell Index Assessment

The cell proliferation was measured as previously described [38], by RTCA instrument (ACEA Biosciences, San Diego, CA, USA). Cells were seeded in 16-well E-plates, and the cell index was recorded every 1 h. The cell index was normalized and presented as relative cell index.

### 4.4. Wound Healing Assay

The wound healing-based cell migration assay was performed as previously described [39]. In brief, equal number of HeLa cells were seeded into dishes, and a wound was scratched using a 200-µL pipette tip when the confluence of cells reached 100%. Subsequently, cells were washed with DPBS thrice to get rid of cell debris, then renewed with fresh MEM medium containing 2% FBS and cultured for another 24 h. After incubation, migrated cells were photographed by a phase contrast microscope. The scratched wound area was analyzed by Image J software (version 1.5.3). Wound healing rate was the ratio of the difference between the wound area at two time points (0 h and 24 h) over the initial scratched area (0 h).

### 4.5. Protein Extraction and Immunoblot Analysis

Cells were washed with ice-cold PBS, then scraped from the surface of the culturing dishes and collected by centrifugation. Total protein lysates were extracted by RIPA lysis buffer containing protease inhibitors and phosphatase inhibitors. Then, the Bradford assay was used to estimate protein concentrations. Protein samples were loaded onto 10% SDS-PAGE gels and transferred to the PVDF membranes. Membranes were blocked with 5% nonfat milk in Tris-Buffered Saline containing 0.05% Tween 20 (TBST) for 1.5 h and then incubated overnight with the primary antibodies at 4 °C. Antibodies against GFP (1:1000, sc-8334, Santa Cruz, CA, USA), hypusine (1:1000, ABS1064, Millipore, Temecula, CA, USA), and β-actin (1:20000, A5441, Sigma-Aldrich) were used in our study. On the next day, after washing three times with 1×TBST, membranes were incubated for 1.5 h with secondary antibodies at room temperature. Protein signals were visualized by Enhanced Chemiluminescent Western Blotting Detection Reagents (GE Healthcare) under a Chemiluminescent Imaging System (Tanon 5200, Tanon Corporation, Minhang, Shanghai, China). The full membranes are shown in Figures S3 and S4.

### 4.6. Protein Structure Prediction and Structural Bioinformatic Analysis

A hierarchical approach, the Deep-learning based Iterative Threading ASSEmbly Refinement (D-I-TASSER) server, was used to predict the protein structure of the EIF5A1, EIF5AL1, and EIF5A1 mutants (EIF5A1-R36W, EIF5A1-T45A, EIF5A1-R109P, EIF5A1-R36W&T45A, EIF5A1-R36W&R109P, and EIF5A1-T45A&R109P) (https://zhanggroup.org/D-I-TASSER, accessed on 9 January 2023) [17]. The model confidence is represented by estimated TM-score (eTM-score), calculated based on the significance of threading template alignments, contact map satisfaction rate, mean absolute error between the distance of the model and the distance of AttentionPotential, and convergence of D-I-TASSER simulations. eTM-score is typically in the range of [0, 1], with a higher eTM-score signifying higher model confidence. AlphaFold Protein Structure Database (https://alphafold.ebi.ac.uk, accessed on 9 January 2023) is a public and extensive database of high-precision protein structure prediction. Supported by AlphaFold v2.0 of DeepMind for covering the structural coverage of the known protein sequence space [15,16].

### 4.7. Statistical Analysis

Immunoblot experiment results were analyzed by Gel Pro Analyzer 4.0 software; other experiment results were analyzed by GraphPad Prism 7 software, with a two-tailed *t*-test for data comparison with *p* < 0.05 considered statistically significant.

## 5. Conclusions

EIF5A1 and EIF5AL1, although there are only three AA differences, their effects on the proliferation and migration ability of HeLa cells are different. The cell proliferation and migration ability of HeLa cells with EIF5AL1 overexpression was significantly decreased compared with EIF5A1. Among the six EIF5A1 mutants, EIF5A1-R36W&T45A and EIF5A1-R36W&R109P decreased the cell proliferation and migration ability of HeLa cells as EIF5AL1. Results of the hypusine modification level and protein stability of EIF5A1, EIF5AL1 and EIF5A1 mutant proteins showed that the hypusine modification was only detectable in EIF5A1 and EIF5A1-T45A proteins, and these two proteins are more stable, indicating that the two arginine residues (arginine 36 and arginine 109) are important for hypusine formation and protein stability of EIF5A1.

## Data Availability

All data generated during this study are included in the main manuscript and Supplementary Materials.

## Supplementary Materials

The supporting information can be downloaded at: https://www.mdpi.com/article/10.3390/ijms24076067/s1.
