# Supplementary material for: Comparison of Human Eukaryotic Translation Initiation Factors 5A1 and 5AL1: Identification of Amino Acid Residues Important for EIF5A1 Lysine 50 Hypusination and Its Protein Stability
Source: Int J Mol Sci. 2023 Mar 23;24(7):6067. doi: 10.3390/ijms24076067 (PMC10093921; doi:10.3390/ijms24076067)
Supplement: Supplementary file 1 [file ijms-24-06067-s001.zip › ijms-2166303-supplementary.pdf]

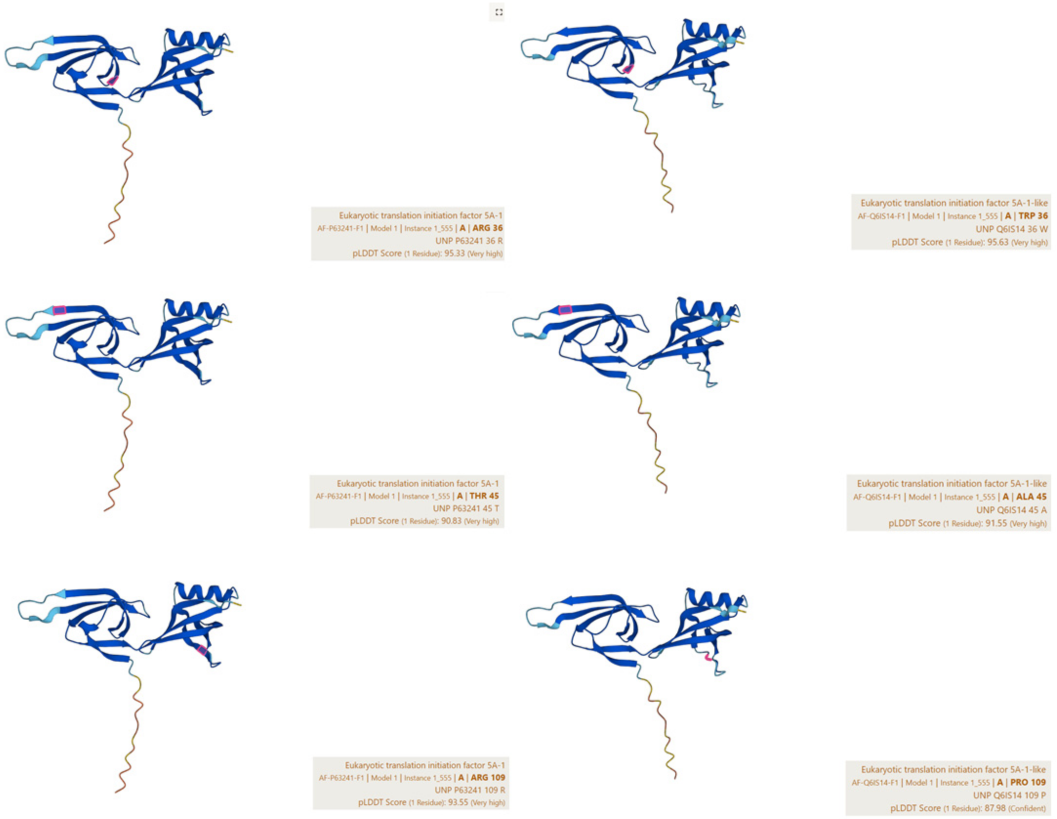

Figure S1. Through the AlphaFold Protein Structure Database (https://alphafold.ebi.ac.uk/, accessed on 9 January 2023), we obtained the AlphaFold protein structures of EIF5A1 (UniProt: P63241) and EIF5AL1 (UniProt: Q6IS14). The red part on each structure represents the position of the amino acid at residue 36, 45, or 109.

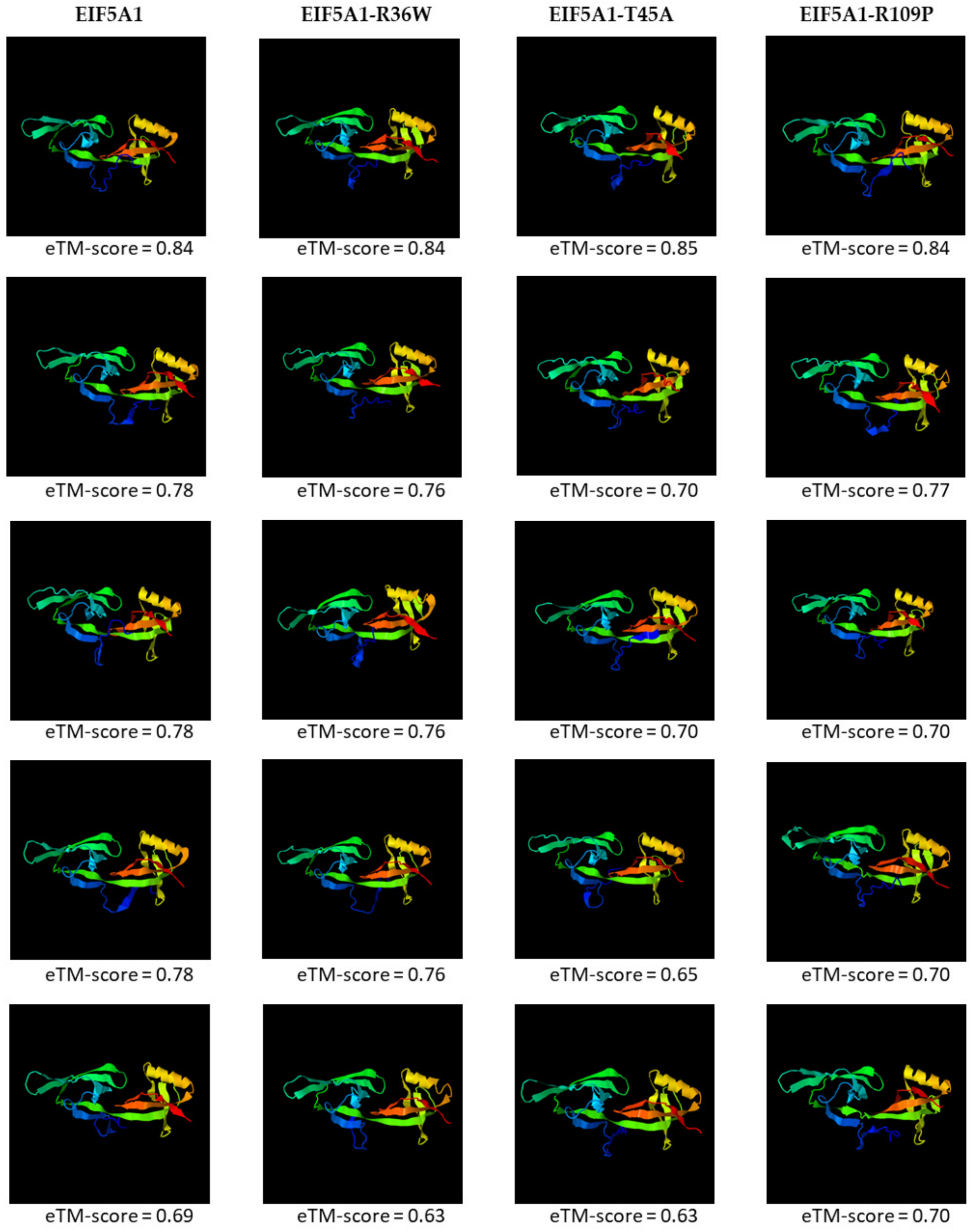

EIF5A1
EIF5A1-R36W
EIF5A1-T45A
EIF5A1-R109P
eTM-score = 0.84
eTM-score = 0.84
eTM-score = 0.85
eTM-score = 0.84
eTM-score = 0.78
eTM-score = 0.76
eTM-score = 0.70
eTM-score = 0.77
eTM-score = 0.78
eTM-score = 0.76
eTM-score = 0.70
eTM-score = 0.70
eTM-score = 0.78
eTM-score = 0.76
eTM-score = 0.65
eTM-score = 0.70
eTM-score = 0.69
eTM-score = 0.63
eTM-score = 0.63
eTM-score = 0.70

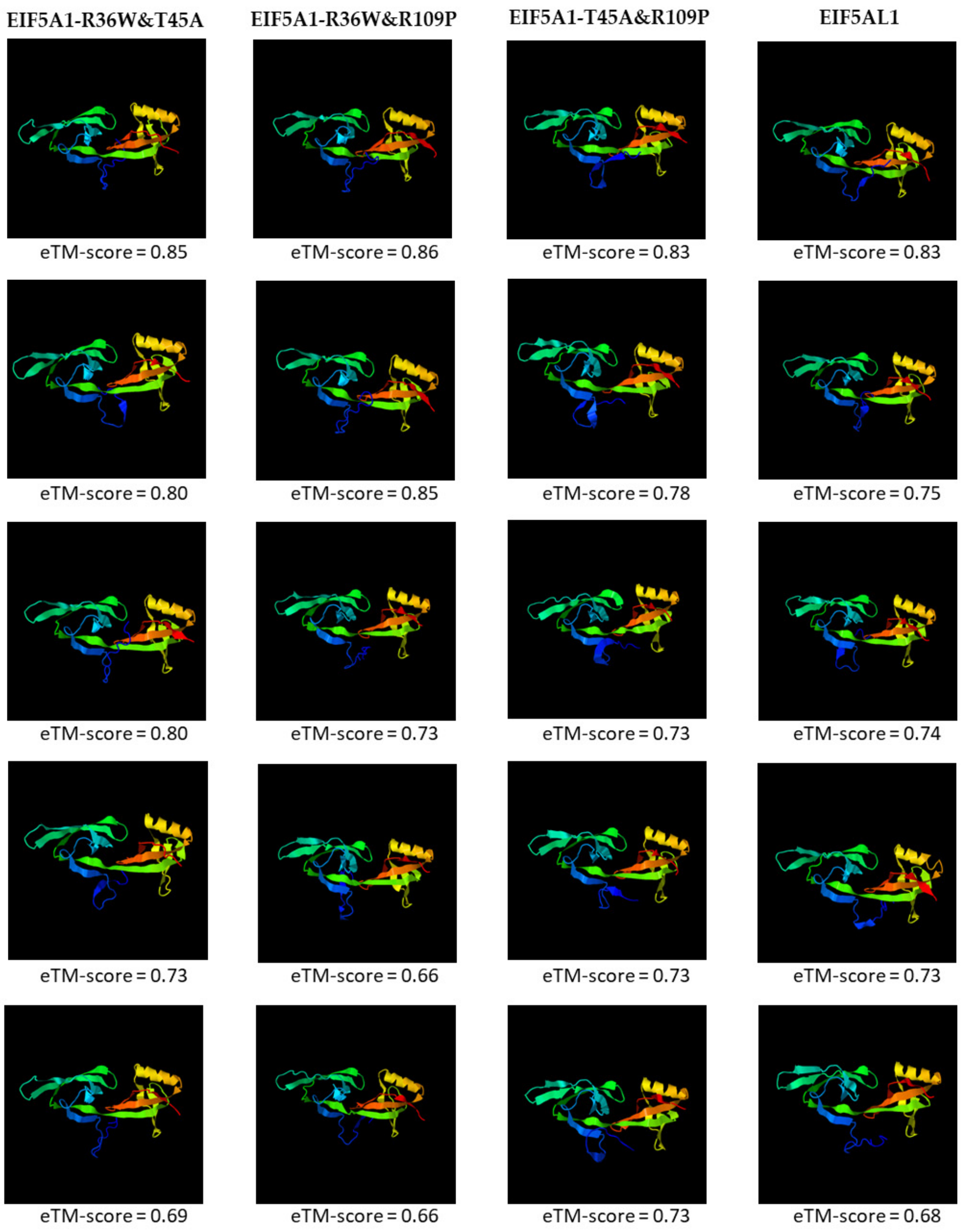

Figure S2. Top five models predicted by D-I-TASSER server (https://zhanggroup.org/D-I-TASSER, accessed on 9 January 2023) based on the amino acid sequences of the EIF5A1, EIF5AL1, and EIF5A1 mutant proteins (EIF5A1-R36W, EIF5A1-T45A, EIF5A1-R109P, EIF5A1-R36W&T45A, EIF5A1-R36W&R109P, and EIF5A1-T45A&R109P). The model confidence is represented by estimated TM-score (eTM-score), calculated based on the significance of threading template alignments, contact map satisfaction rate, mean absolute error between the distance of the model and the distance of AttentionPotential, and convergence of D-I-TASSER simulations. eTM-score is typically in the range of [0, 1], with higher eTM-score signifying higher model confidence.

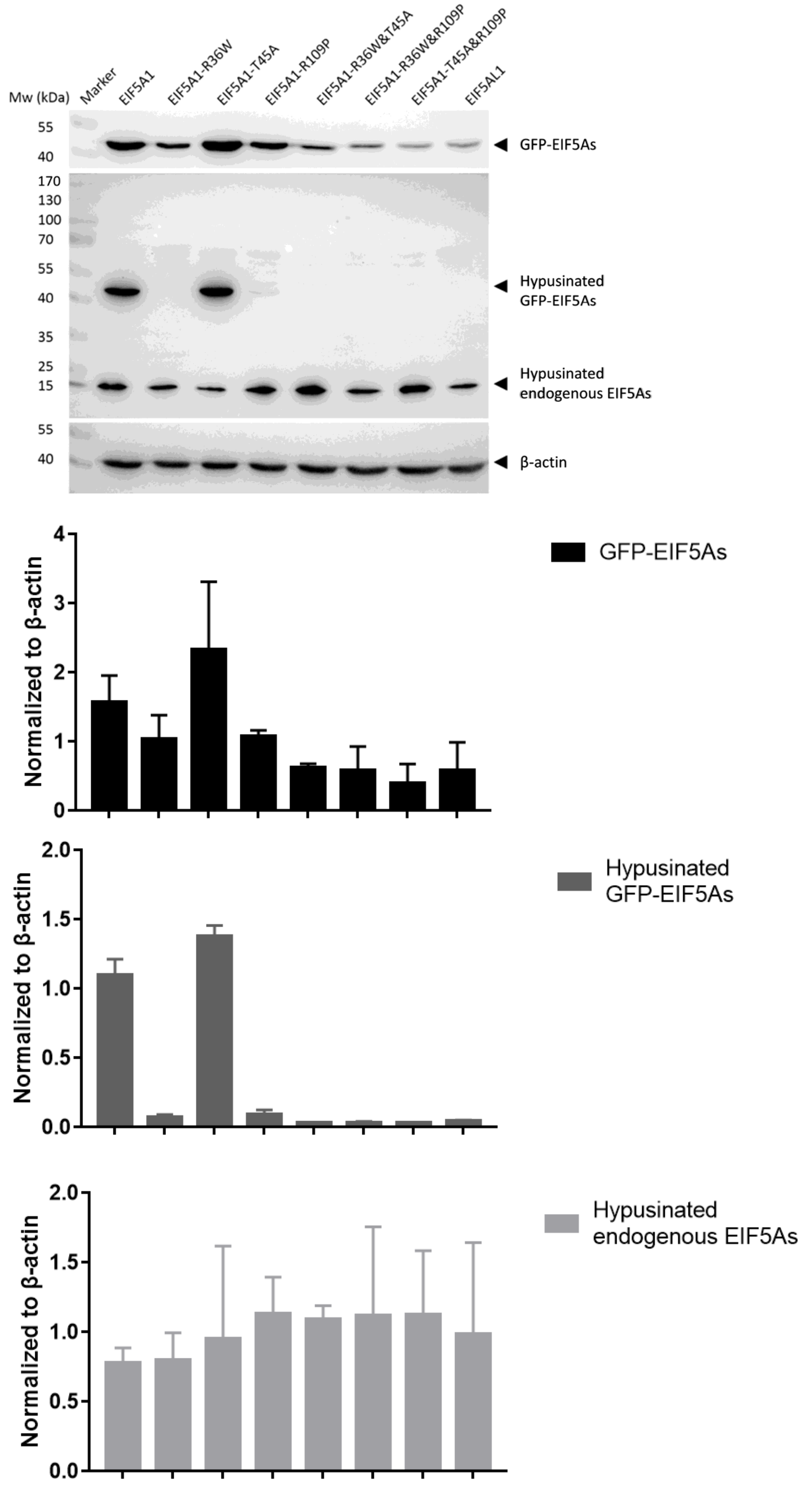

Figure S3. The hypusine modification levels of EIF5A1, EIF5AL1, and EIF5A1 mutant proteins. HeLa cells were transfected with EIF5A1, EIF5AL1, and EIF5A1 mutant plasmids, cells were harvested and lysed at 48 h after transfection. The proteins were extracted, and hypusine modification levels of EIF5A1, EIF5AL1, as well as EIF5A1 mutant proteins were determined by immunoblot analysis. Signals were quantified and normalized to β-actin.

A

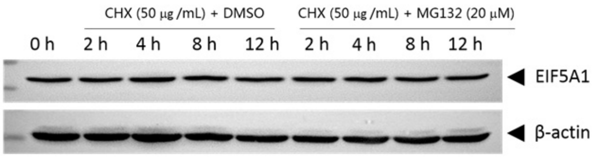

Time (h)	CHX + DMSO		CHX + MG132	
	Gray-scale value	SD	Gray-scale value	SD
0	1	0	1	0
2	0.882058	0.106831	0.816051667	0.152887
4	1.169935	0.305641	0.956614667	0.102654
8	1.128189667	0.200309	1.017477667	0.510483
12	0.942497333	0.118323	0.977494	0.289536

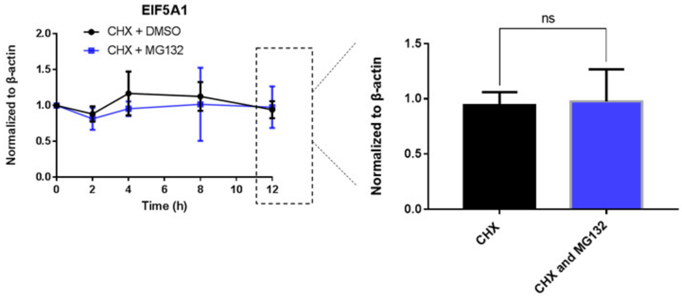

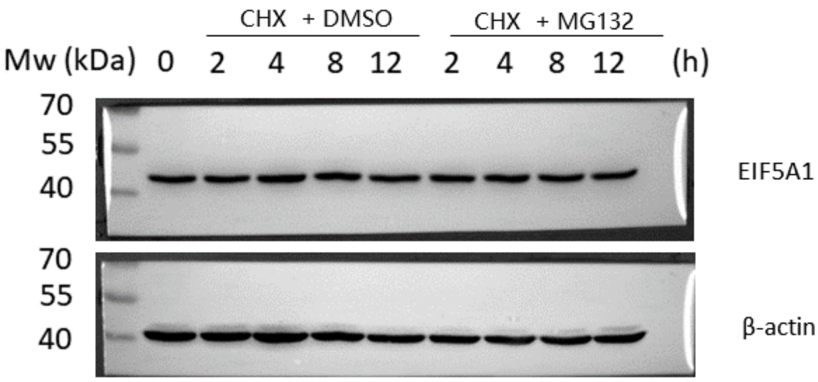

B

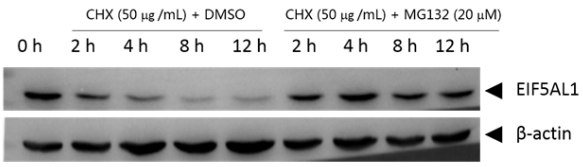

Time (h)	CHX + DMSO		CHX + MG132	
	Gray-scale value	SD	Gray-scale value	SD
0	1	0	1	0
2	0. 4696085	0. 064519	0. 6320475	0. 006973
4	0. 195258	0. 018713	0. 619512	0. 261296
8	0. 0734415	0. 000412	0. 62795	0. 22087
12	0. 179879	0. 00882	0. 5794145	0. 127771

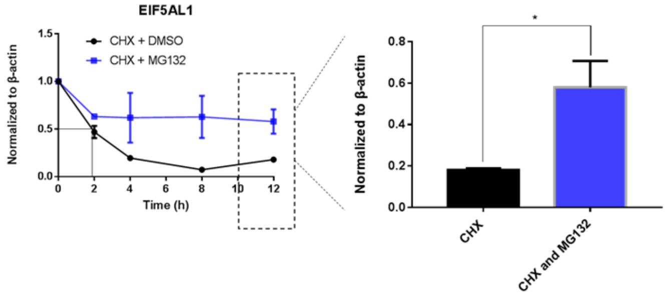

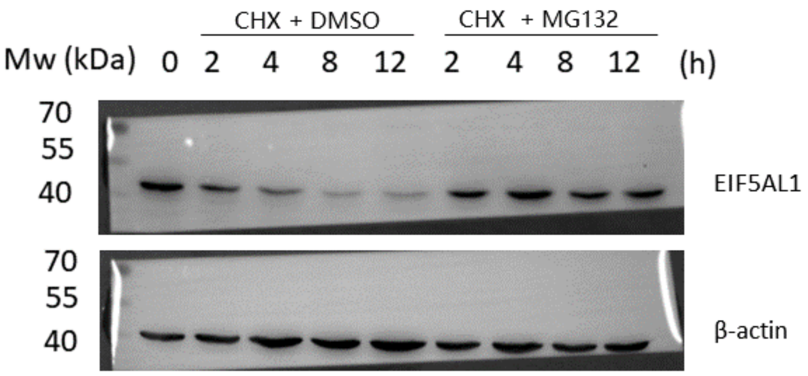

C

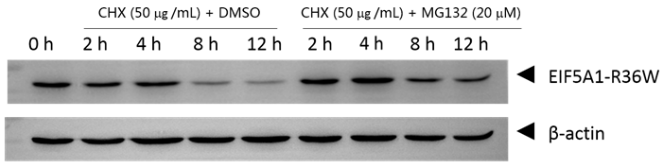

Time (h)	CHX + DMSO		CHX + MG132	
	Gray-scale value	SD	Gray-scale value	SD
0	1	0	1	0
2	0.780932	0.430777	0.9971835	0.025902
4	0.955995	0.02337	1.214434	0.064514
8	0.292516	0.052993	0.787019	0.140741
12	0.146782	0.037989	0.84876	0.213886

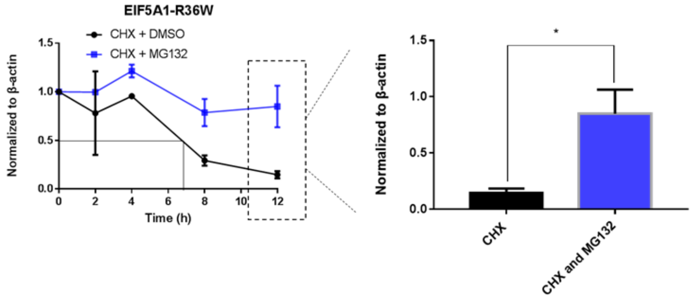

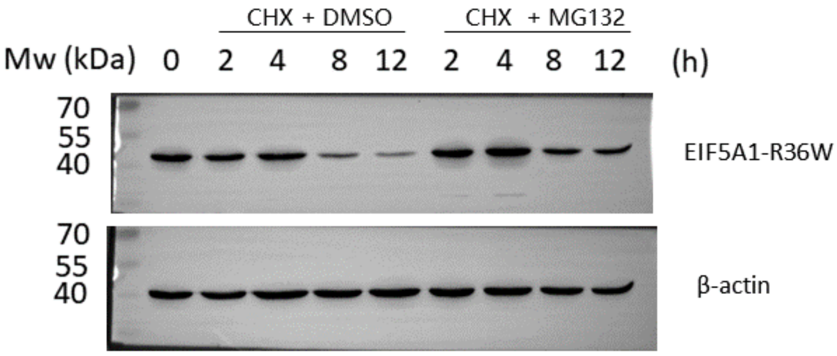

D

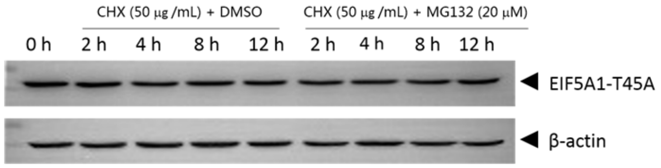

Time (h)	CHX + DMSO		CHX + MG132	
	Gray-scale value	SD	Gray-scale value	SD
0	1	0	1	0
2	1. 068077	0. 467604	1. 068519	0. 226396
4	0. 8795535	0. 360417	1. 082629	0. 362013
8	0. 986438	0. 398605	1. 23375	0. 337395
12	1. 039838	0. 454568	1. 100628	0. 020007

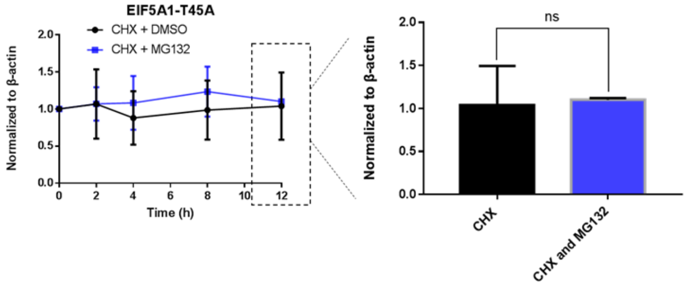

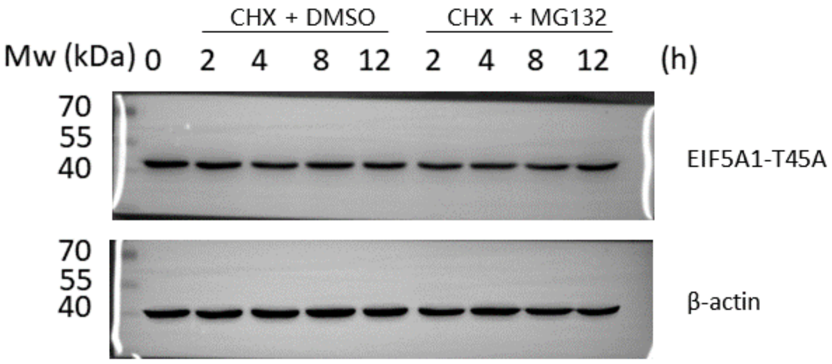

E

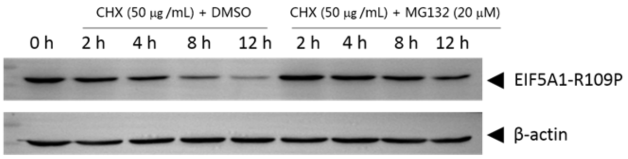

Time (h)	CHX + DMSO		CHX + MG132	
	Gray-scale value	SD	Gray-scale value	SD
0	1	0	1	0
2	0. 789273	0. 276507	1. 0146735	0. 176439
4	0. 5848935	0. 147479	0. 906623	0. 065344
8	0. 3395315	0. 203746	0. 894721	0. 299178
12	0. 0742185	0. 008267	0. 682922	0. 057427

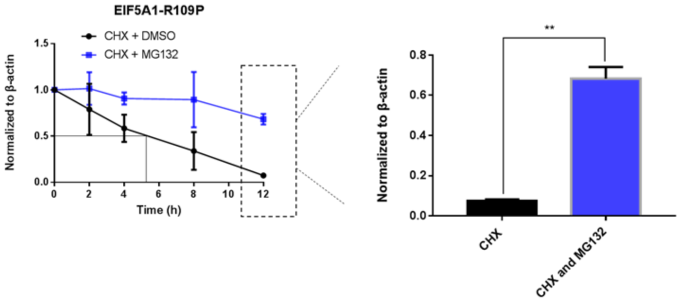

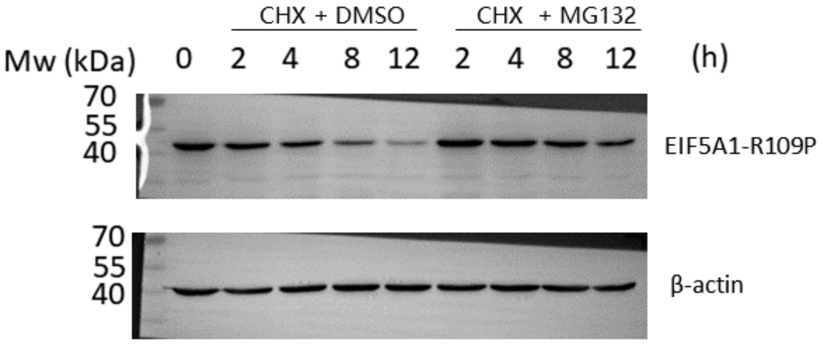

F

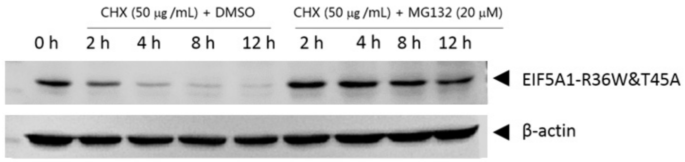

处理时间（h）	CHX + DMSO		CHX + MG132	
	Gray-scale value	SD	Gray-scale value	SD
0	1	0	1	0
2	0.6343875	0.079198	0.9534685	0.45612
4	0.2448215	0.054372	1.138086	0.353904
8	0.09418	0.006563	0.8313765	0.23847
12	0.099453	0.018921	0.7079675	0.154192

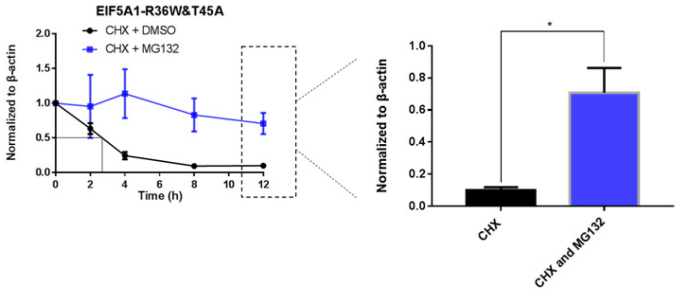

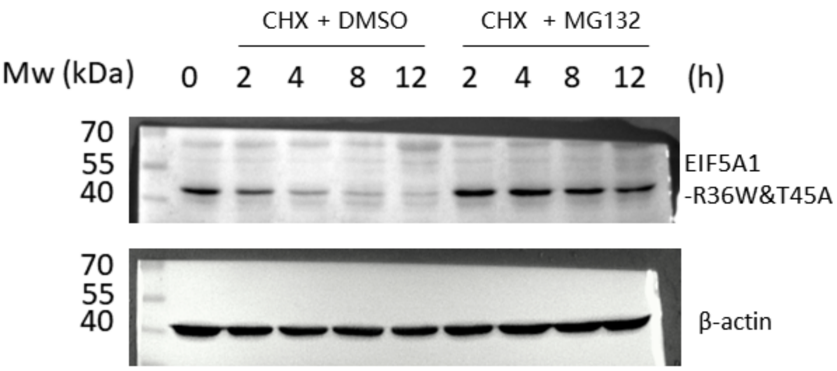

G

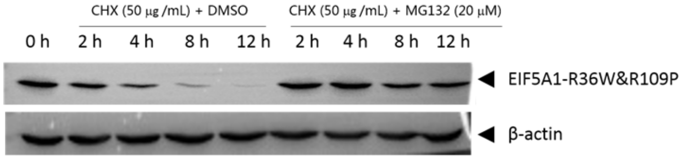

Time (h)	CHX + DMSO		CHX + MG132	
	Gray-scale value	SD	Gray-scale value	SD
0	1	0	1	0
2	0. 61891	0. 031567	0. 7648195	0. 001604
4	0. 2847425	0. 004662	0. 7706555	0. 001418
8	0. 0909365	0. 011412	0. 5205295	0. 071045
12	0. 025922	0. 020911	0. 4524465	0. 067251

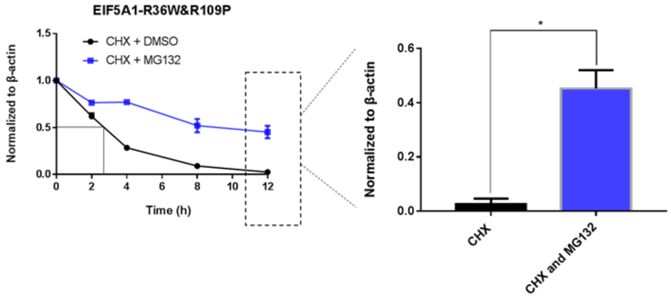

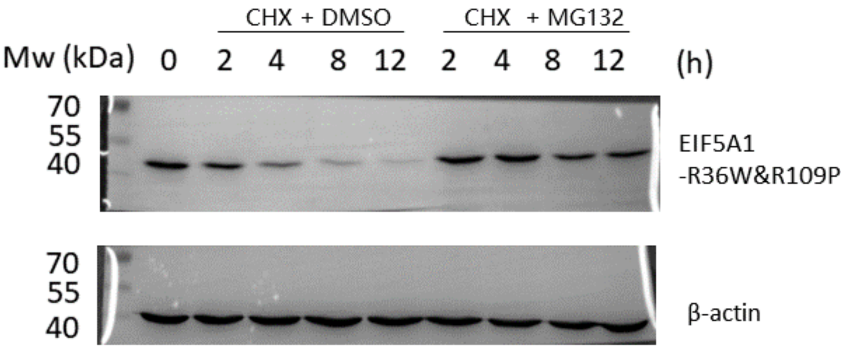

H

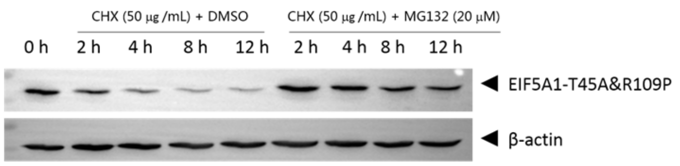

Time (h)	CHX + DMSO		CHX + MG132	
	Gray-scale value	SD	Gray-scale value	SD
0	1	0	1	0
2	0. 5119	0. 041173	1. 112067	0. 05625
4	0. 262667	0. 027243	0. 8300795	0. 001681
8	0. 1538505	0. 007198	0. 772841	0. 039482
12	0. 10692	0. 009467	0. 620696	0. 02463

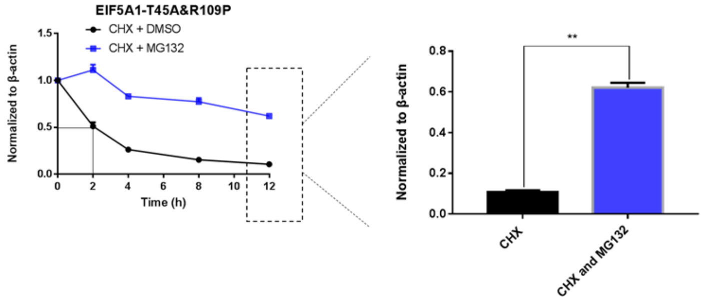

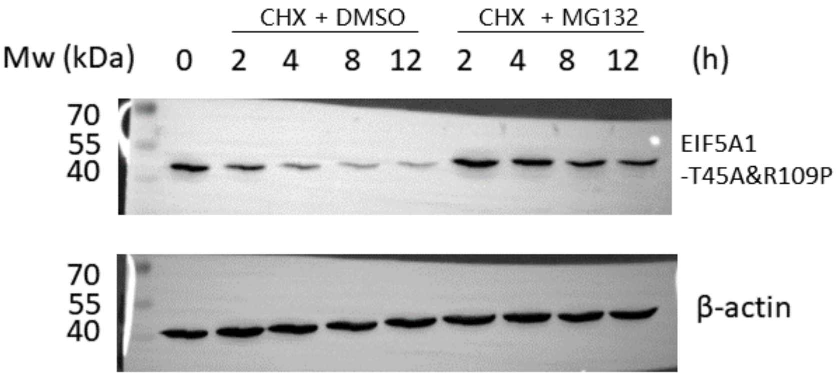

Figure S4. HeLa cells were seeded into 10 cm cell culture dishes for 24 h, transfected with EIF5A1, EIF5AL1, and EIF5A1 mutant plasmids, and the cells were averaged to nine 6 cm cell culture dishes at 24 h post-transfection. After 24 h, four of these cells were treated individually with CHX, and four others with CHX and MG132. Cells were harvested after the indicating time points, and the abundance of EIF5A1, EIF5AL1, as well as EIF5A1 mutant proteins were determined by immunoblot analysis using GFP antibody. β-actin was used as the loading control. (A) EIF5A1 protein abundance. (B) EIF5AL1 protein abundance. (C) EIF5A1-R36W protein abundance. (D) EIF5A1-T45A protein abundance. (E) EIF5A1-R109P protein abundance. (F) EIF5A1-R36W&T45A protein abundance. (G) EIF5A1-R36W&R109P protein abundance. (H) EIF5A1-T45A&R109P protein abundance. ns, $p > 0.05$; *, $p \leq 0.05$; **, $p \leq 0.01$.

Table S1. Primers for constructing EIF5A1, EIF5AL1, and the EIF5A1 mutants.

Gene Name	Primer Name	Primer Sequence (5' to 3')	Length (nt.)	Tm (℃)
EIF5A1	Sense	cggaattcgccaccatggcagatgacttggacttcga	37	68.9
	Anti-sense	cgggatcccgttttgccatggccttgattgca	32	67.0
EIF5AL1	Sense	gaattcgccaccatggcagatgatttggactt	32	63.1
	Anti-sense	ggatcccgttttgccatggccttgattgcaa	31	64.4
**EIF5A1 is the template**				
EIF5A1-R36W	Sense	cgatcttacatggccagcctttgagcaccac	31	78.98
	Anti-sense c106t_	gtggtgctcaaaggctggccatgtaagatcg	31	78.98
EIF5A1-T45A	Sense	cttgccagtcttcgaagcagacatctcgacgatct	35	79.27
	Anti-sense a133g_	agatcgtcgagatgtctgcttcgaagactggcaag	35	79.27
EIF5A1-R109P	Sense	cgaaggtcctctggtacctccccgc	25	78.38
	Anti-sense g326c_	gcggggaggtaccagaggaccttcg	25	78.38
**EIF5AL1 is the template**				
EIF5A1-R36W&T45A	Sense	cgaaggtcctctcttacctccccgctgtcc	30	78.30
	Anti-sense c325a_c326g_	ggacagcggggaggtaagagaggaccttcg	30	78.30
EIF5A1-R36W&R109P	Sense	cttgccagtcttcgaagtagacatctccacgatct	35	79.27
	Anti-sense g133a_	agatcgtggagatgtctacttcgaagactggcaag	35	79.27
EIF5A1-T45A&R109P	Sense	cgatcttacatggcctgcctttgagcaccac	31	78.98
	Anti-sense t106a_	gtggtgctcaaaggcaggccatgtaagatcg	31	78.98

Table S2. Sequencing results of the EIF5A1 mutants (The first nucleotide is indicated by the blue arrow, the mutation site by the red arrow, and the last nucleotide by the green arrow).

Gene Name	Sequence
EIF5A1-R36W	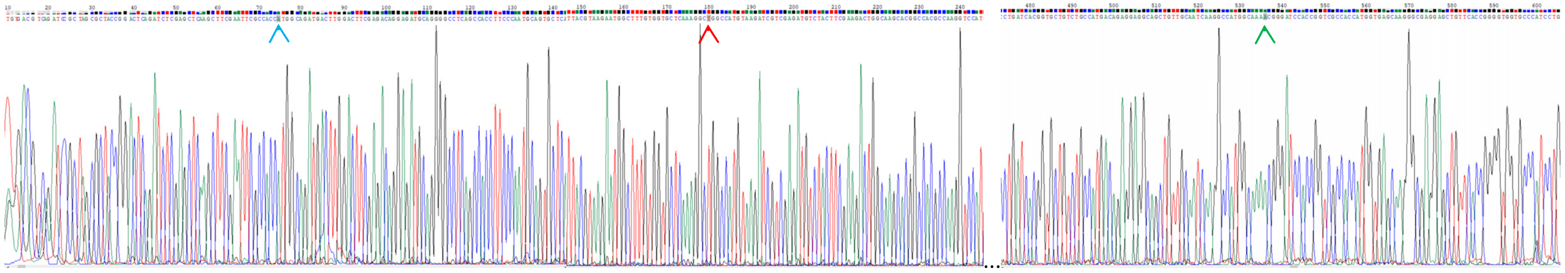
EIF5A1-T45A	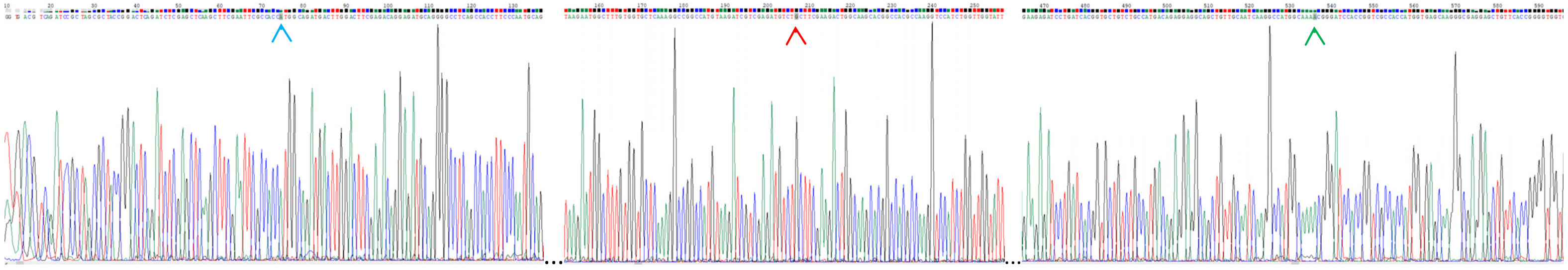
EIF5A1-R109P	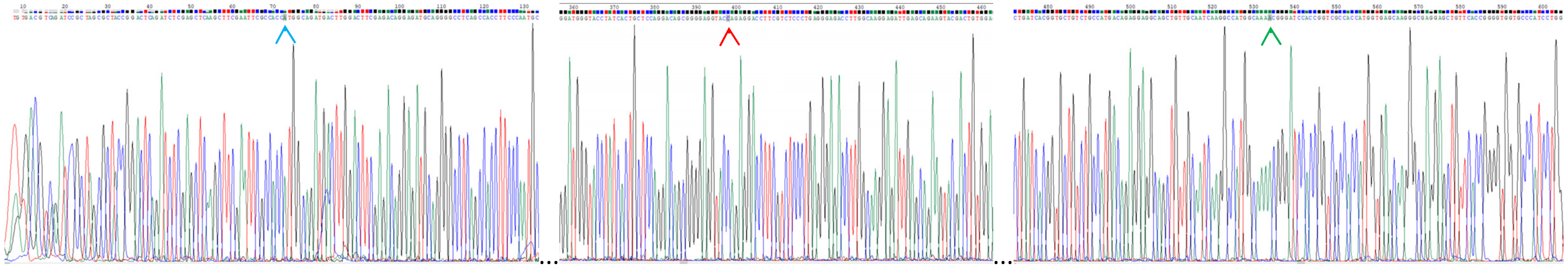

EIF5A1-R36W&T45A	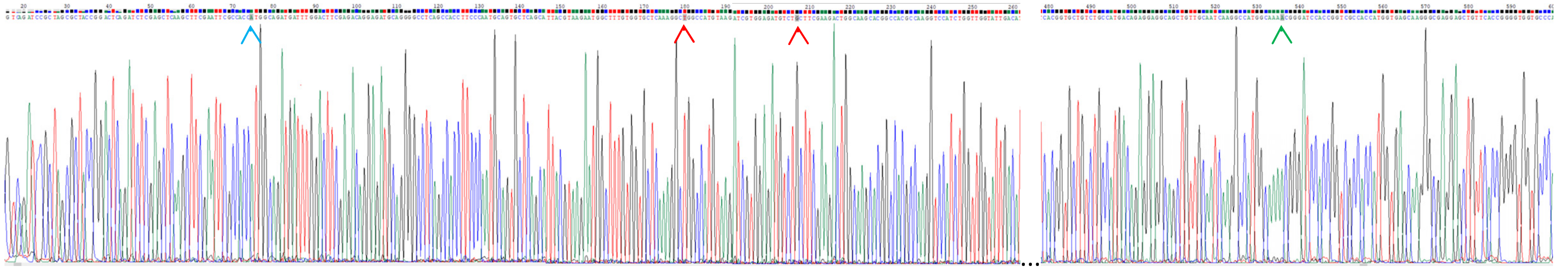
EIF5A1-R36W&R109P	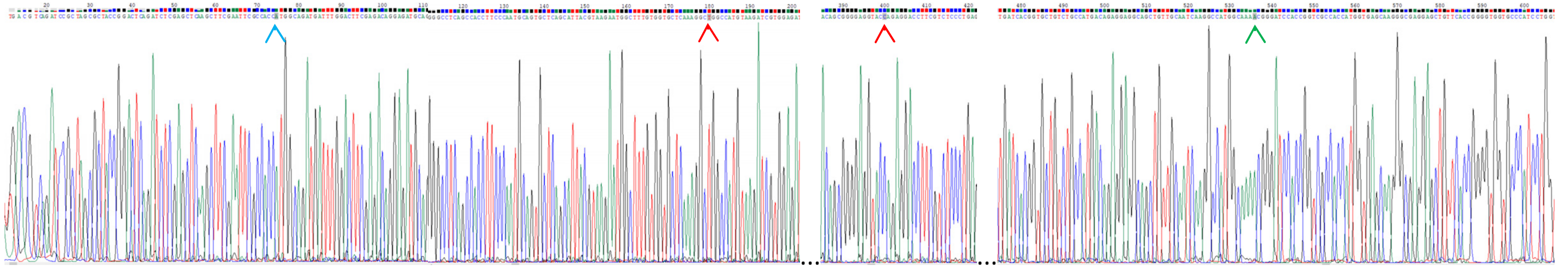
EIF5A1-T45A&R109P	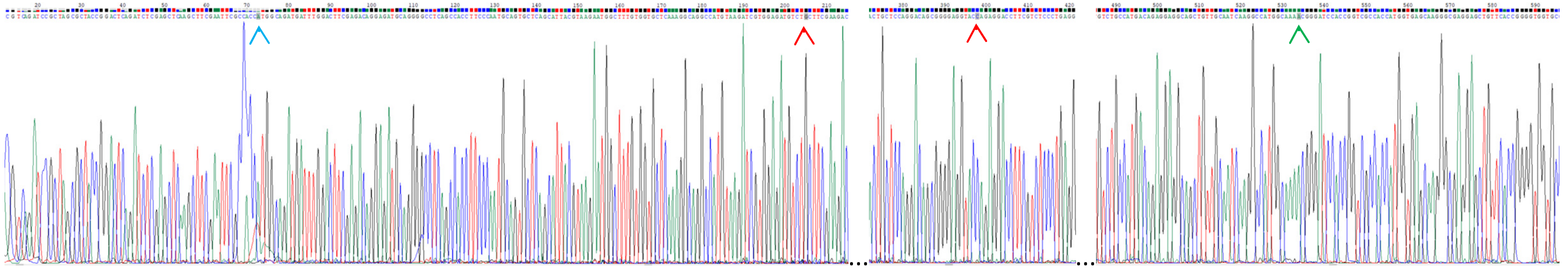
